# Platelet-to-neutrophil ratio and in-hospital mortality in pneumonia patients receiving glucocorticoid therapy: a multicenter retrospective cohort study

**DOI:** 10.3389/fmed.2025.1731128

**Published:** 2026-01-12

**Authors:** Junxin Lin, Ruitao Chen, Shangbo Xu, Weihan Lin

**Affiliations:** 1 Department of Respiratory and Critical Care Medicine, Jieyang People's Hospital, Jieyang, China; 2 Department of Cardiology, Jieyang People's Hospital, Jieyang, China

**Keywords:** glucocorticoids, mortality, platelet-to-neutrophil ratio, pneumonia, prognosis

## Abstract

**Background:**

Pneumonia remains a leading cause of global mortality. Patients receiving glucocorticoid therapy represent a particularly vulnerable subgroup due to therapy-induced immunosuppression, which complicates diagnosis and worsens prognosis. The platelet-to-neutrophil ratio (PNR), a composite hematologic index, has shown prognostic utility in various diseases, but its value in glucocorticoid-treated pneumonia patients remains unexplored.

**Methods:**

This multicenter retrospective cohort study utilized data from 686 hospitalized pneumonia patients receiving systemic glucocorticoids, extracted from the Dryad database. The association between admission PNR and all-cause in-hospital mortality at 30 and 90 days was assessed using Cox proportional hazards models. Receiver operating characteristic curves were used to evaluate predictive performance.

**Results:**

In-hospital mortality within both 30 and 90 days decreased significantly with increasing PNR tertiles (Tertile 1 vs. Tertile 3: 37.55% vs. 9.61% for 30-day; 43.67% vs. 10.92% for 90-day). In fully adjusted models, each standard deviation increase in PNR was associated with a 50% reduction in 30-day in-hospital mortality [Hazard Ratio (HR) = 0.497, 95% CI 0.262–0.942] and a 58% reduction in 90-day in-hospital mortality (HR = 0.416, 95% CI 0.218–0.793). Compared to the lowest tertile, the highest PNR tertile was associated with a 76% lower risk of 30-day mortality (HR = 0.235, 95% CI 0.107–0.513) and an 82% lower risk of 90-day mortality (HR = 0.184, 95% CI 0.085–0.398). PNR demonstrated superior predictive ability (areas under the curve for 30-day: 0.707; 90-day: 0.713) compared to platelet or neutrophil count alone. Subgroup analysis revealed stronger associations in patients receiving high-dose glucocorticoids and those with diabetes or hypertension.

**Conclusion:**

A lower PNR at admission is independently associated with increased short-term mortality in pneumonia patients receiving glucocorticoids. PNR, as a readily available biomarker, may facilitate early risk stratification and identify high-risk patients who could benefit from more aggressive management.

## 1 Introduction

Pneumonia remains a leading global cause of morbidity and mortality, accounting for more than 2.18 million deaths in 2021 alone (1). Despite its substantial toll, community-acquired pneumonia is seldom viewed as a high-priority public-health issue; yet roughly one-third of patients die within a year of hospital discharge (2). Worldwide, up to 18% of patients hospitalized for community-acquired pneumonia have at least one immunosuppressive risk factor (2). Patients receiving long-term or high-dose glucocorticoid therapy represent a vulnerable subgroup, as such treatment induces a state of profound immunosuppression (3). This not only dramatically increases their susceptibility to a broad spectrum of opportunistic and conventional pulmonary pathogens but also masks typical signs of infection, often leading to delayed diagnosis and advanced disease at presentation (3–6). Previous studies have shown that up to 74% of patients on chronic glucocorticoid therapy develop pneumonia within the first year of treatment (7), with mortality rates ranging from 26 to 45%, depending on disease severity and underlying comorbidities (3, 7). Despite advances in antimicrobial therapy and supportive care, these sobering figures underscore the persistent clinical challenges and the urgent need for simple, readily available prognostic biomarkers to facilitate early risk stratification and guide more aggressive monitoring and treatment in this high-risk population.

In recent years, hematologic indices derived from routine complete blood counts have emerged as promising prognostic tools in various clinical settings (8, 9). Among these, the platelet-to-neutrophil ratio (PNR) has gained attention for its potential to reflect the inflammation responses and thrombosis (10, 11). Previous studies have demonstrated the prognostic utility of PNR in malignancies (12, 13), myocardial infarction (14), and stroke (15). More recently, a study by Li et al. highlighted its role in differentiating sepsis from neonatal pneumonia (16), suggesting its broader applicability in infectious diseases. Despite these promising findings, the prognostic value of PNR in pneumonia patients receiving glucocorticoid therapy remains unexplored. Given the complex interplay between glucocorticoid-induced immunosuppression, dysregulated neutrophil responses, and thrombocytopenia in severe infections, PNR may serve as a composite marker reflecting both inflammatory burden and immune dysregulation.

Therefore, this study aimed to investigate the association between PNR and all-cause in-hospital mortality in hospitalized pneumonia patients undergoing glucocorticoid therapy, using a multicenter retrospective cohort.

## 2 Methods

### 2.1 Data source and study population

This retrospective cohort study utilized data from the Dryad digital repository (https://datadryad.org/), originally published by Li et al. (17). The original study was a multicenter investigation conducted across six secondary and tertiary academic hospitals in China between January 2013 and December 2017 (7). To ensure consistency, a common study protocol and a single formatted case report form (CRF) were used across all centers. Investigators from all sites received standardized training prior to data collection, and completed CRFs were reviewed for quality control. Ethical approval for the original study was granted by the Ethics Committee of the China-Japan Friendship Hospital (Approval No. 2015–86), and all data were anonymized prior to public release.

**Figure 1:**
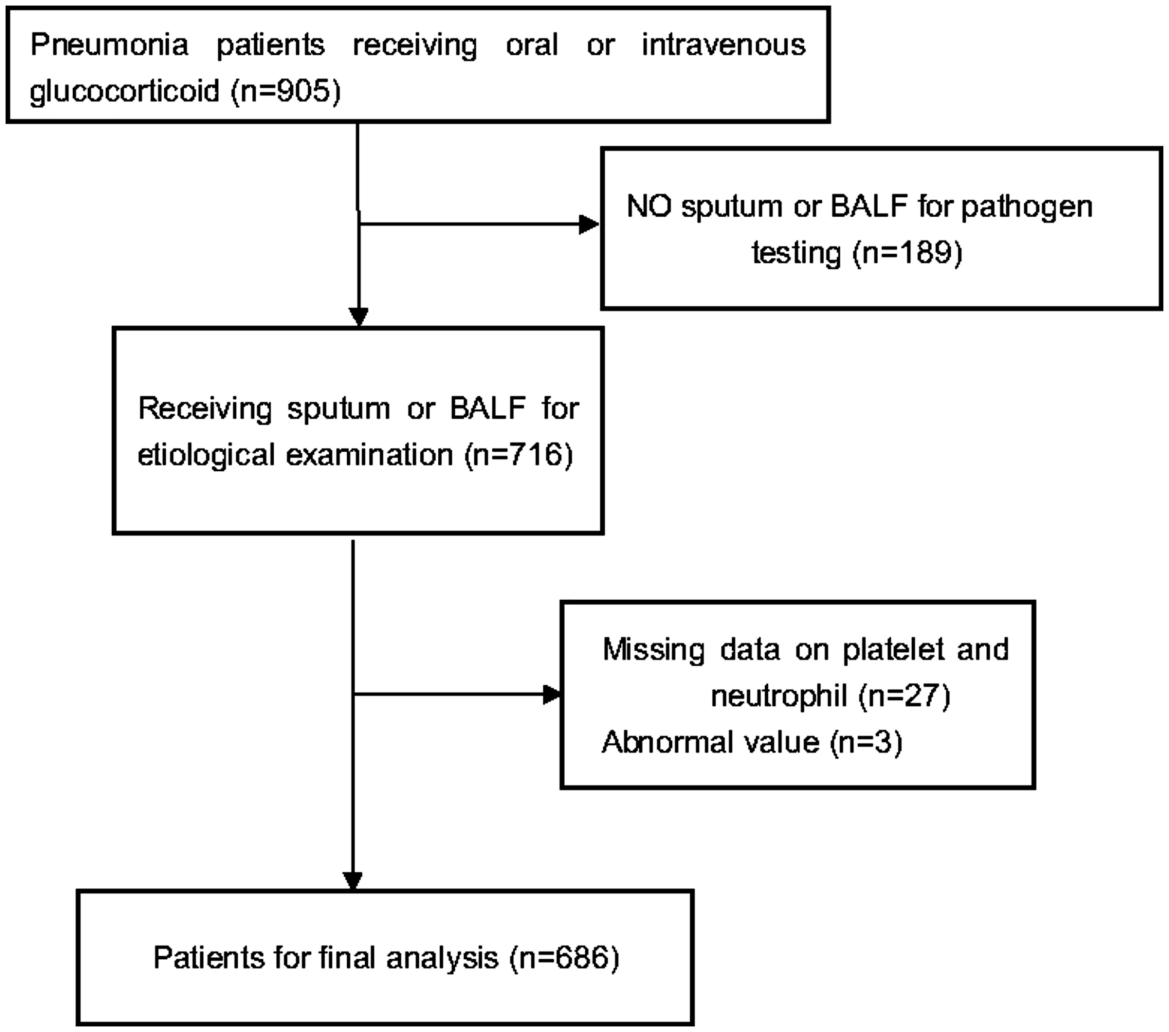
Flow chart.

The original cohort included 716 patients who developed pneumonia while receiving systemic glucocorticoids. Pneumonia was diagnosed according to the American Thoracic Society and Infectious Diseases Society of America guidelines, based on radiographic evidence of new pulmonary infiltrates plus clinical signs of infection (18, 19). The diagnosis required radiographic evidence (chest X-ray or CT) of new pulmonary infiltrates, plus the presence of one or more clinical signs of infection (e.g., fever, cough, sputum production, dyspnea, leukocytosis/leukopenia). Inclusion criteria were: (1) receipt of oral or intravenous glucocorticoids before admission, (2) diagnosis of pneumonia at admission or during hospitalization, and (3) age ≥16 years. Patients lacking complete platelet (PLT) or neutrophil (NEUT) records, as well as those whose PNR fell outside the physiologically plausible range, were excluded. The final analytic sample consisted of 686 participants (Figure 1).

### 2.2 Data extraction and variable definition

The following variables were extracted from the dataset: demographic information (age, sex, self-reported smoking status and alcohol consumption); clinical presentation data (admission body temperature, heart rate, blood pressure and a full spectrum of comorbidities such as diabetes, hypertension and connective-tissue disease); laboratory results including complete blood counts (PLT, NEUT, lymphocytes, hemoglobin), albumin, lactate dehydrogenase, alanine aminotransferase, total bilirubin, blood urea nitrogen and serum creatinine; treatment details covering any use of high-dose glucocorticoids (≥30 mg/day prednisolone equivalent) and the cumulative glucocorticoid dose administered during hospitalization; and, finally, the primary outcomes of all-cause in-hospital mortality within 30 and 90 days of admission, ascertained from the inpatient medical records.

PNR was calculated as the ratio of PLT count (×10^9^/L) to NEUT count (×10^9^/L) measured from the first routine blood sample obtained at hospital admission. Patients were categorized into tertiles based on PNR values for descriptive and comparative analyses: Tertile 1 ≤ 19.83; Tertile 2 19.83–36.79; Tertile 3 ≥ 36.80. PNR was also analyzed as a continuous variable to assess dose–response relationships.

### 2.3 Statistical analysis

Continuous variables were expressed as mean ± standard deviation or median (interquartile range) based on distribution, and categorical variables as frequencies (percentages). Differences across PNR tertiles were assessed using ANOVA, Kruskal–Wallis tests, or chi-square tests as appropriate.

The associations between PNR and in-hospital mortality were evaluated using Cox proportional hazards models, with results reported as hazard ratios (HRs) and 95% confidence intervals (CIs). Both continuous (per standard deviation [SD] increment) and categorical (by tertile) analyses were conducted. Multivariable models were adjusted for potential confounders selected a priori based on clinical relevance and previous literature: age, sex, smoking, alcoholism, high-dose glucocorticoid use, laboratory parameters, and key comorbidities. Three models were constructed: Model 1: unadjusted; Model 2: adjusted for ex, age, smoking status, alcohol use, and recorded comorbidity; Model 3: further adjusted for temperature, heart rate, systolic blood pressure, diastolic blood pressure, pneumonia severity index (PSI), high-dose glucocorticoids, lymphocytes, hemoglobin, albumin, lactate dehydrogenase, alanine aminotransferase, total bilirubin, and creatinine. The proportional hazards assumption for the Cox models (Model 3) was confirmed using Schoenfeld residual tests (global test *p* > 0.05). Multicollinearity among covariates was assessed by calculating the variance inflation factor (VIF); all VIF values were below 2.5, indicating no substantial collinearity. Restricted cubic splines (RCS) with four knots placed at the 5th, 35th, 65th, and 95th percentiles of the PNR distribution were used to examine potential non-linearity in the relationship between the PNR and in-hospital mortality.

Kaplan–Meier survival curves were plotted to visualize cumulative survival across PNR tertiles, with differences assessed using the log-rank test. The discriminative performance of admission PNR for 30- and 90-day mortality was evaluated with receiver operating characteristic (ROC) curves; areas under the curve (AUC) were compared against those of PLT count alone and NEUT count alone by DeLong’s test. Subgroup analyses were performed to assess potential effect modification by age, sex, high-dose glucocorticoid use, diabetes, and hypertension. Interaction terms were included in regression models, and *p*-values for interaction were reported.

All analyses were conducted using SPSS version 27.0 and R software version 4.0.5. A two-sided *p*-value <0.05 was considered statistically significant.

## 3 Results

### 3.1 Characteristics of the population by PNR

A total of 686 pneumonia patients who received glucocorticoid treatment were enrolled in this study (Figure 1). Following admission, all-cause in-hospital mortality was recorded at 22.16% (*n* = 152) for 30 days and 25.66% (*n* = 176) for 90 days, respectively. Participants were classified into three groups according to their PNR values; their initial clinical profiles are summarized in Table 1.

**Table 1:** Baseline characteristics of participants stratified by platelet to neutrophil ratio.

Variables	Tertile 1 (*n* = 229)	Tertile 2 (*n* = 228)	Tertile 3 (*n* = 229)	*P*-value
Male, *n* (%)	93 (40.61)	105 (46.05)	128 (55.90)	0.004
Age ≥60 years, *n* (%)	127 (55.46)	109 (47.81)	123 (53.71)	0.230
Smoke				0.125
Never	159 (69.43)	163 (71.49)	179 (78.17)	
Former	58 (25.33)	59 (25.88)	41 (17.90)	
Current	12 (5.24)	6 (2.63)	9 (3.93)	
Alcoholism, *n* (%)	27 (11.79)	19 (8.33)	11 (4.80)	0.026
Diseases, *n* (%)				
COPD	26 (11.35)	38 (16.67)	36 (15.72)	0.229
Asthma	4 (1.75)	3 (1.32)	9 (3.93)	0.139
ILD	97 (42.36)	114 (50.00)	102 (44.54)	0.240
Hypertension	82 (35.81)	73 (32.02)	82 (35.81)	0.617
CHD	32 (13.97)	28 (12.28)	24 (10.48)	0.522
CHF	11 (4.80)	5 (2.19)	3 (1.31)	0.060
Diabetes mellitus	56 (24.45)	57 (25.00)	56 (24.45)	0.988
Nephrotic syndrome	37 (16.16)	28 (12.28)	24 (10.48)	0.181
CRF	23 (10.04)	13 (5.70)	17 (7.42)	0.216
CTD	118 (51.53)	117 (51.32)	116 (50.66)	0.981
Cerebrovascular disease	14 (6.11)	18 (7.89)	17 (7.42)	0.746
Pneumonia and treatment				
Temperature (°C)	37.10 (36.50, 38.40)	37.00 (36.50, 38.00)	36.90 (36.50, 37.60)	< 0.001
Heart rate(bpm)	92.00 (80.00, 108.00)	86.50 (80.00, 104.00)	87.00 (80.00, 99.00)	0.007
Systolic pressure (mmhg)	122.00 (112.00, 135.00)	120.00 (110.00, 133.00)	121.00 (111.00, 134.00)	0.291
Diastolic pressure (mmhg)	77.00 (68.00, 83.00)	75.00 (66.00, 81.00)	75.00 (68.00, 80.00)	0.587
Pneumonia severity index	87.00 (68.00, 116.00)	75.00 (55.00, 93.25)	70.00 (53.00, 89.00)	< 0.001
High-dose glucocorticoids, *n* (%)	100 (43.67)	83 (36.40)	61 (26.64)	0.001
Accumulated dose of glucocorticoids, methylprednisolone (g)	3.30 (1.78, 6.59)	3.98 (2.16, 8.20)	4.59 (2.27, 11.76)	0.018
Blood test				
NEUT (×109/L)	10.09 (6.86, 13.41)	7.14 (5.53, 9.79)	4.02 (2.77, 5.29)	< 0.001
LYM (×109/L)	0.75 (0.41, 1.24)	0.80 (0.50, 1.31)	1.04 (0.69, 1.56)	< 0.001
HGB (g/L)	110.00 (91.00, 129.00)	117.00 (102.00, 131.00)	111.00 (94.00, 126.00)	0.006
PLT (×109/L)	131.00 (85.00, 178.00)	201.00 (152.00, 255.50)	214.00 (175.00, 278.00)	< 0.001
ALB (g/L)	31.00 (27.30, 35.05)	32.25 (29.22, 36.15)	34.60 (30.00, 38.00)	< 0.001
LDH (U/L)	400.00 (281.25, 591.00)	348.00 (229.00, 509.00)	257.00 (196.00, 387.00)	< 0.001
ALT (U/L)	29.50 (17.00, 54.95)	23.00 (16.00, 36.50)	21.00 (13.00, 37.00)	0.001
TBIL (U/L)	11.69 (7.64, 18.32)	10.13 (6.80, 13.93)	7.79 (5.98, 11.10)	0.001
CRE (mmol/L)	69.50 (51.90, 115.70)	62.55 (49.65, 81.30)	64.00 (51.80, 84.35)	0.022
30-day in-hospital mortality	86 (37.55)	44 (19.30)	22 (9.61)	< 0.001
90-day in-hospital mortality	100 (43.67)	51 (22.37)	25 (10.92)	< 0.001

Data are shown as mean ± standard deviation (SD) or median (IQR) for continuous variables and proportions (%) for categorical variables.

COPD, chronic obstructive pulmonary disease; ILD, interstitial lung disease; CHD, coronary heart disease; CRF, chronic renal failure; CTD, connective tissue disease; NEUT, neutrophils; LYM, lymphocytes; HGB, hemoglobin; PLT, platelets; ALB, albumin; LDH, lactate dehydrogenase; ALT, alanine aminotransferase; TBIL, total bilirubin; CRE, creatinine.

Patients in the lowest PNR tertile were more likely to be female and had a higher prevalence of alcoholism. On admission they exhibited greater clinical severity: temperature, heart rate and pneumonia severity index were all significantly elevated. NEUT, lactate dehydrogenase, alanine aminotransferase, total bilirubin, and creatinine were highest in Tertile 1, while lymphocyte count, hemoglobin, PLT, and albumin were lowest. Glucocorticoid exposure differed across tertiles: high-dose methylprednisolone was used most often in Tertile 1, while cumulative glucocorticoid dose increased with PNR. No significant differences were observed across tertiles for age ≥ 60 years, smoking status, blood pressure or any recorded comorbidity.

### 3.2 The in-hospital mortality in different PNR groups

**Figure 2:**
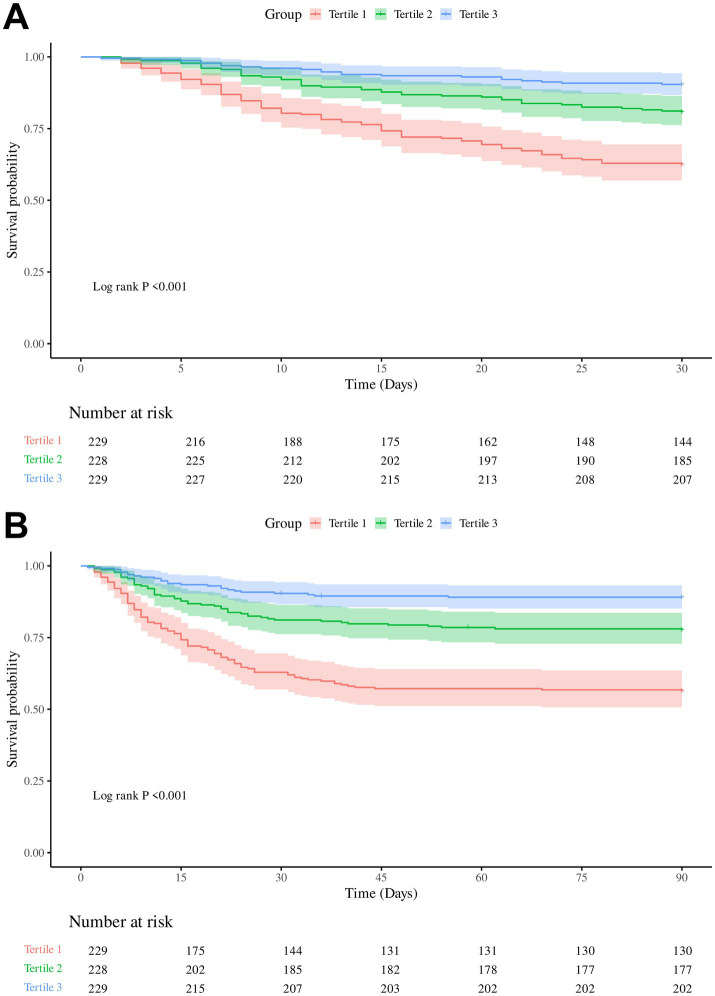
Kaplan–Meier survival curves for 30-day and 90-day in-hospital mortality according to platelet-to-neutrophil ratio (PNR) tertiles. **(A)** Cumulative survival for 30-day mortality. **(B)** Cumulative survival for 90-day mortality.

Both 30-day and 90-day mortality rates decreased significantly with increasing PNR: from 37.55 and 43.67% in Tertile 1 to 9.61 and 10.92% in Tertile 3, respectively (Table 1). Kaplan–Meier analysis demonstrated a significant inverse association between PNR levels and survival, with progressively lower 30-day and 90-day survival probabilities observed in descending PNR tertiles (Log-rank *p* < 0.001) (Figure 2).

### 3.3 Relationship between PNR and 30-day in-hospital mortality

**Figure 3:**
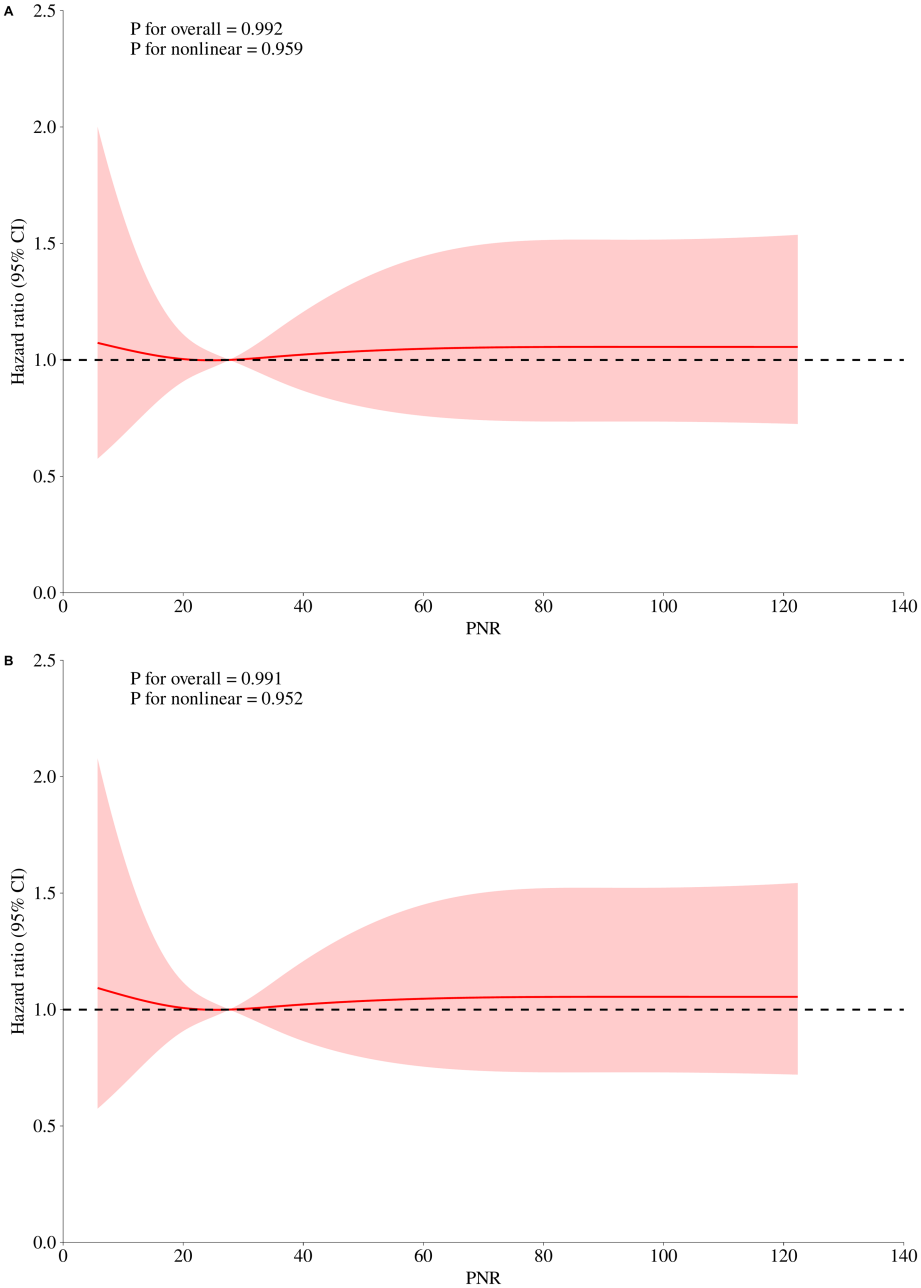
Restricted cubic spline analyses of the association between admission platelet-to-neutrophil ratio (PNR) and mortality in pneumonia patients receiving glucocorticoids: **(A)** 30-day in-hospital mortality; **(B)** 90-day in-hospital mortality.

The results of the initial univariate Cox regression for 30-day mortality in pneumonia patients were summarized in Supplementary Table S1. Subsequent multivariate Cox regression (Table 2) showed a consistent and inverse association between PNR and 30-day mortality. Per SD increase in PNR, the risk of death fell by 66% in the unadjusted model (HR = 0.337, 95% CI 0.224–0.507, *p* < 0.001) and remained 50% lower after full adjustment (HR = 0.497, 95% CI 0.262–0.942, *p* = 0.032). Compared with the lowest tertile, patients in the middle and highest PNR thirds had stepwise reductions in mortality (Tertile 2: adjusted HR = 0.513, 95% CI 0.294–0.893, *p* = 0.018; Tertile 3: adjusted HR = 0.235, 95% CI 0.107–0.513, *p* < 0.001), with P-for-trend < 0.001 across all models. The linearity of the continuous association was formally assessed using RCS (Figure 3). For 30-day in-hospital mortality, the relationship between PNR and the mortality was monotonically decreasing and approximately linear across its observed range (*p* for nonlinearity = 0.959).

**Table 2:** Association between PNR and in-hospital mortality.

PNR	Model 1		Model 2		Model 3	
	OR (95%CI)	*P*	OR (95%CI)	*P*	OR (95%CI)	*P*
30-day mortality						
PNR (per 1 SD)	0.337 (0.224–0.507)	<0.001	0.347 (0.229–0.526)	<0.001	0.497 (0.262–0.942)	0.032
PNR (Tertile)						
Tertile 1	Ref		Ref		Ref	
Tertile 2	0.452(0.315–0.651)	<0.001	0.457 (0.316–0.662)	<0.001	0.513 (0.294–0.893)	0.018
Tertile 3	0.215(0.135–0.344)	<0.001	0.224 (0.139–0.361)	<0.001	0.235 (0.107–0.513)	<0.001
*P* for trend		<0.001		<0.001		<0.001
90-day mortality						
PNR (per 1 SD)	0.311(0.211–0.458)	<0.001	0.326(0.220–0.484)	<0.001	0.416(0.218–0.793)	0.008
PNR (Tertile)						
Tertile 1	Ref		Ref		Ref	
Tertile 2	0.440(0.314–0.617)	<0.001	0.444(0.315–0.627)	<0.001	0.492(0.291–0.833)	0.008
Tertile 3	0.203(0.131–0.315)	<0.001	0.215(0.138–0.336)	<0.001	0.184(0.085–0.398)	<0.001
*P* for trend		<0.001		<0.001		<0.001

Model 1: unadjusted.

Model 2: sex, age, smoking status, alcohol use, and recorded comorbidity.

Model 3: adjusted for variables in Model 2 plus temperature, heart rate, systolic blood pressure, diastolic blood pressure, pneumonia severity index, high-dose glucocorticoids, lymphocytes, hemoglobin, albumin, lactate dehydrogenase, alanine aminotransferase, total bilirubin, and creatinine.

PNR, platelet to neutrophil ratio; HR, hazard ratio; CI, confidence interval; Ref., reference.

### 3.4 Relationship between PNR and 90-day in-hospital mortality

Univariate Cox regression results for 90-day mortality in pneumonia patients are presented in Supplementary Table S2. Each SD increment in PNR conferred a 69% lower hazard in the crude analysis (HR = 0.311, 95% CI 0.211–0.458, *p* < 0.001) and a 58% reduction after full covariate control (HR = 0.416, 95% CI 0.218–0.793, *p* = 0.008). Categorically, the adjusted risk of death was approximately halved in Tertile 2 (HR = 0.492, 95% CI 0.291–0.833, *p* = 0.008) and fell by >80% in Tertile 3 (HR = 0.184, 95% CI 0.085–0.398, *p* < 0.001) relative to the lowest PNR group, with significant trend tests throughout. Similarly, for 90-day in-hospital mortality, the RCS analysis confirmed a linear relationship between PNR and the mortality (*p* for nonlinearity = 0.952) (Figure 3).

### 3.5 The ability of PNR to predict in-hospital mortality

**Figure 4:**
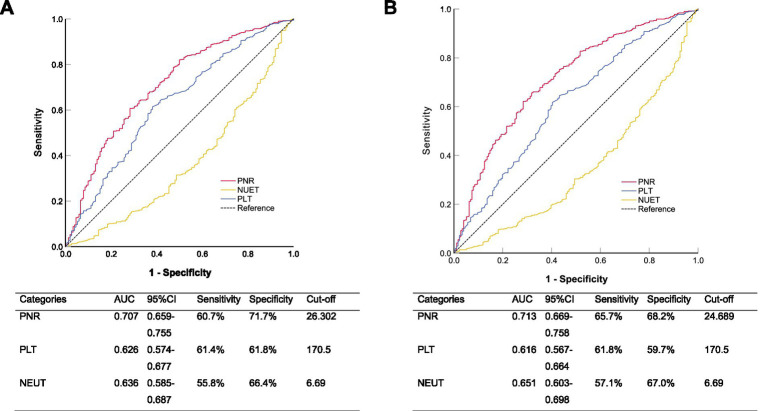
Receiver operating characteristic curves comparing the ability of platelet-to-neutrophil ratio (PNR), platelet count, and neutrophil count to predict **(A)** 30-day and **(B)** 90-day all-cause in-hospital mortality.

To evaluate the predictive performance of PNR, PLT, and NEUT for 30-day and 90-day mortality, we conducted ROC curve analysis (Figure 4). For 30-day mortality, the AUC for PNR was 0.707 (95% CI 0.659–0.755), with a sensitivity of 60.7% and a specificity of 71.7% at the optimal cut-off value of 26.302, outperforming both PLT (AUC = 0.626, 95% CI 0.574–0.677; sensitivity 61.4%, specificity 61.8%) and NEUT (AUC = 0.636, 95% CI 0.585–0.687; sensitivity 55.8%, specificity 66.4%). Similarly, for 90-day mortality, PNR again demonstrated the highest predictive ability with an AUC of 0.713 (95% CI 0.669–0.758), a sensitivity of 65.7%, and a specificity of 68.2% at a cut-off of 24.689, compared to PLT (AUC = 0.616, 95% CI 0.567–0.664; sensitivity 61.8%, specificity 59.7%) and NEUT (AUC = 0.651, 95% CI 0.603–0.698; sensitivity 57.1%, specificity 67.0%). These results indicated that PNR was a stable and superior predictor for both 30-day and 90-day mortality compared to PLT and NEUT alone. For predicting 30-day in-hospital mortality, the PNR achieved an AUC of 0.707 (95% CI 0.659–0.755), which was numerically higher than but not statistically different from the AUC of PSI score (0.690, 95% CI 0.644–0.736; DeLong’s test, *p* = 0.582). A similar pattern was observed for 90-day mortality (PNR AUC: 0.713 vs. PSI AUC: 0.704; *p* = 0.757).

### 3.6 Subgroup analysis

**Figure 5:**
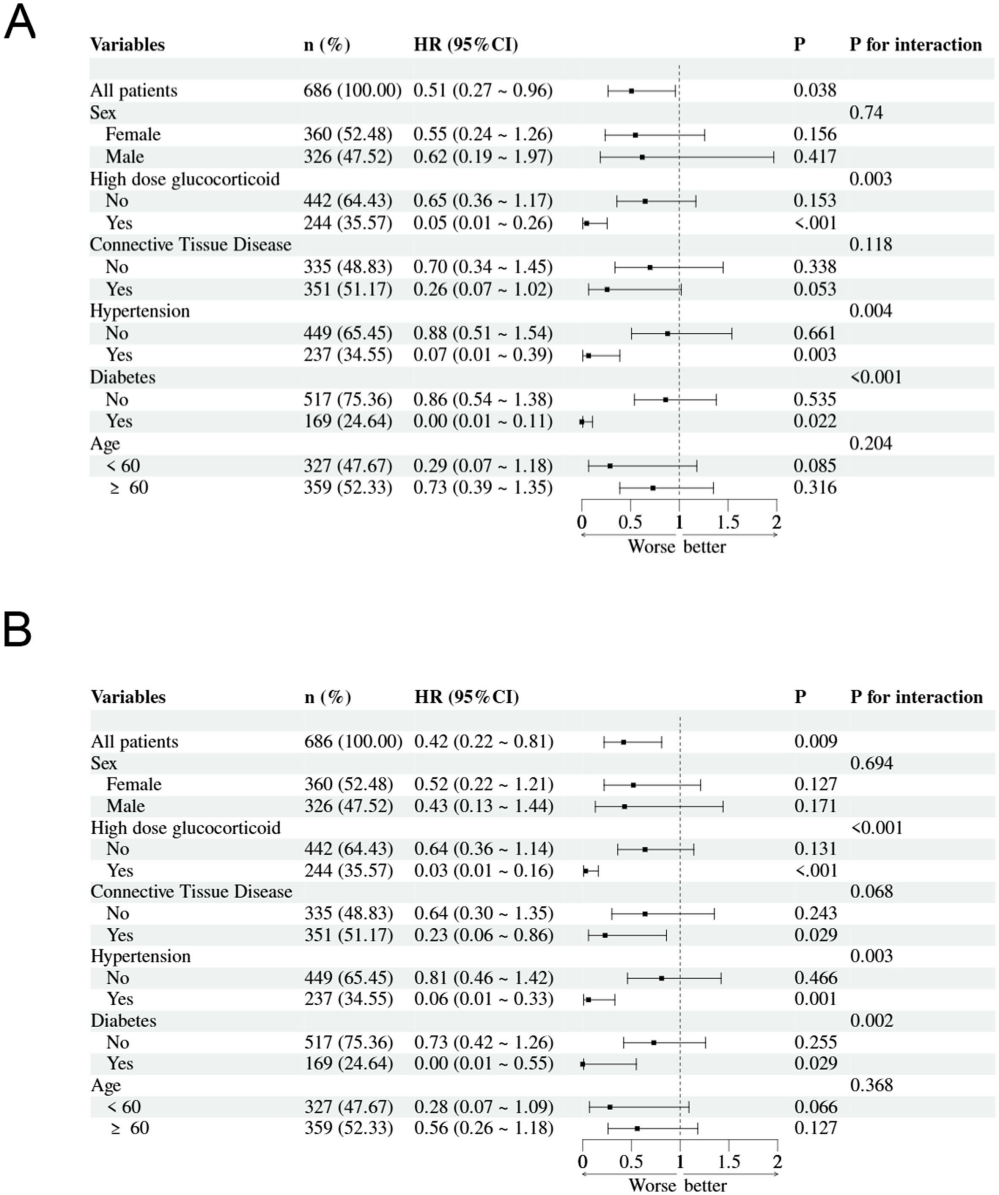
Subgroup analyses of the association between Platelet-to-Neutrophil Ratio (PNR) and in-hospital mortality: **(A)** 30-day mortality and **(B)** 90-day mortality.

We performed subgroup analyses to assess the association between PNR levels and in-hospital mortality risk among pneumonia patients receiving glucocorticoid treatment at 30-day and 90-day follow-up (Figure 5). Significant interactions were observed for high-dose glucocorticoid therapy (30-day: P-interaction = 0.003; 90-day: P-interaction < 0.001), with markedly lower mortality risk in patients receiving high-dose glucocorticoids. Significant interactions were also found for hypertension (30-day: P-interaction = 0.004; 90-day: P-interaction = 0.003) and diabetes (30-day: P-interaction < 0.001; 90-day: P-interaction = 0.002), showing stronger protective effects of PNR in patients with these comorbidities. A marginally significant interaction was observed for connective tissue disease at 90-day follow-up (*P*-interaction = 0.068), with enhanced protective effect in affected patients. No significant interactions were found for sex or age groups in either timepoint.

### 3.7 Sensitivity analyses

To assess the robustness of the association between PNR and mortality beyond the primary tertile-based categorization, we conducted sensitivity analyses using alternative cut-off values. When patients were dichotomized at the median PNR value, those in the lower half had significantly higher 30-day (adjusted HR = 0.371, 95% CI 0.211–0.653, *p* < 0.001) and 90-day (adjusted HR = 0.320, 95% CI 0.186–0.552, *p* < 0.001) in-hospital mortality compared to those in the upper half, after full adjustment (Model 3). Using the Youden-index derived optimal cut-off values (26.302 for 30-day and 24.689 for 90-day mortality, see Section 3.5), patients with a PNR below the cut-off had a significantly increased risk of death (30-day: adjusted HR = 0.298 95% CI 0.167–0.530, *p* < 0.001; 90-day: adjusted HR = 0.287, 95% CI 0.168–0.490, *p* < 0.001) compared to those above the cut-off, after full adjustment.

## 4 Discussion

In this retrospective cohort study utilizing data from the Dryad database, we investigated the prognostic value of the PNR in hospitalized pneumonia patients receiving prolonged glucocorticoid therapy. Our results demonstrated that a lower PNR was strongly and independently associated with increased 30-day and 90-day all-cause in-hospital mortality. Patients in the lowest PNR tertile exhibited significantly higher in-hospital mortality rates (37.55% at 30 days; 43.67% at 90 days) compared to those in the highest tertile (9.61 and 10.92%, respectively). After comprehensive adjustment for confounders, each SD increase in PNR was associated with a 50% reduction in 30-day in-hospital mortality and a 58% reduction in 90-day in-hospital mortality. Furthermore, PNR demonstrated superior predictive performance for both 30-day and 90-day in-hospital mortality compared to PLT count or NEUT count alone, as evidenced by higher AUC values in ROC analysis.

To our knowledge, this is the first study to evaluate PNR as a prognostic marker specifically in pneumonia patients receiving glucocorticoids. Previous investigations have highlighted the utility of PNR in other clinical contexts, including malignancies (12, 13), myocardial infarction (14), and stroke (15), where it often reflects systemic inflammation and immune dysregulation. A recent study by Li et al. also suggested the clinical utility of PNR in differentiating sepsis from neonatal pneumonia, underscoring its role in infection-related outcomes (16). Our findings align with these reports and extend the applicability of PNR to a high-risk immunocompromised population.

The biological plausibility of PNR as a prognostic marker lies in its encapsulation of two critical pathways in severe infection: neutrophil-driven inflammation and platelet-mediated responses, both of which are directly modulated by glucocorticoid therapy. As the main effectors of the innate immune response, NEUT is vital in combating pneumonia by releasing inflammatory cytokines, chemokines, and regulatory cytokines, as well as by directly engulfing and killing pathogens through antimicrobial peptides, proteases, and oxidants (20–22). While this response is essential for host defense, it is also a major driver of collateral tissue damage (21–23). The very proteases and oxidants that neutralize pathogens can inflict significant harm on the alveolar epithelium, exacerbating lung injury (22). The context of glucocorticoid therapy adds a layer of complexity. While these drugs are powerful immunosuppressants that blunt global immunity and heighten vulnerability to opportunistic pathogens, they exert paradoxical and nuanced effects on innate immunity, particularly NEUT. Beyond merely increasing counts, glucocorticoids delay NEUT apoptosis via genomic mechanisms, promoting a sustained state of neutrophilia that may reflect impaired inflammation resolution rather than effective microbial clearance (24, 25). This glucocorticoid induced neutrophilia thus signals a dysregulated, non resolving inflammatory milieu, an aberration precisely mirrored by the NEUT limb of the PNR. Simultaneously, PLT plays a far more complex role than merely mediating coagulation (26, 27). They are active participants in the immune response, functioning as circulating sentinels (27, 28). PLT can directly interact with pathogens, modulate endothelial cell activation, and form aggregates with NEUT. These Platelets-neutrophils aggregates amplify NEUT recruitment and activation, further fueling inflammatory tissue injury. In pneumonia and sepsis, PLT is increasingly consumed at sites of microvascular injury and within forming thrombi, leading to a relative or absolute decline in PLT count (29–31). This thrombocytopenia is a well-established marker of disease severity. The interplay with glucocorticoid therapy is complex and bidirectional. On one hand, corticosteroids may acutely increase PLT count and reactivity; experimental studies show they can promote thrombopoiesis (e.g., by increasing circulating immature PLT counts) and potentially cause PLT demargination (29, 30). On the other hand, their predominant clinical impact in the setting of severe, ongoing infection like pneumonia is often overwhelmed by the profound consumptive thrombocytopenia driven by microvascular injury and immunothrombosis (31–33). Therefore, in our cohort of active pneumonia, a low PNR likely reflects the net outcome where the consumptive loss of PLT outstrips any steroid-driven production increase, occurring alongside a (potentially steroid-exacerbated) dysregulated neutrophilia. Thus, PNR serves as a composite hematologic indicator of a maladaptive host response under iatrogenic immunosuppression, pointing toward a higher risk of organ failure and death.

Moreover, glucocorticoid therapy is known to alter PLT production and function, and may induce lymphopenia while increasing NEUT counts (24, 29). This imbalance may be particularly pronounced in patients with high cumulative steroid exposure, as observed in our Tertile 1 group. The strong interaction between PNR and high-dose glucocorticoid use in our subgroup analysis further supports this integrated pathophysiological mechanism, wherein high-dose steroids may amplify the neutrophilic response while simultaneously failing to compensate for the severe consumptive thrombocytopenia, leading to an even lower PNR and worse prognosis.

Our study introduces PNR as a novel, readily available, and inexpensive prognostic marker for pneumonia patients on glucocorticoids. Unlike more complex scoring systems (34), PNR can be easily derived from routine complete blood counts, making it highly feasible for rapid risk stratification in clinical settings. Its predictive performance surpassed that of isolated PLT or NEUT counts, highlighting the additive value of evaluating their ratio. Notably, the robust predictive power of PNR was quantitatively demonstrated through ROC analysis. PNR achieved an AUC of 0.707 and 0.713 for predicting 30-day and 90-day in-hospital mortality, respectively, outperforming both PLT and NEUT counts alone. This establishes PNR not merely as a statistically significant associate, but as a clinically useful biomarker with good discriminatory ability for identifying high-risk patients upon hospital admission.

Furthermore, our subgroup analyses revealed that the prognostic impact of PNR was not uniform across all patient characteristics. Notably, the association between low PNR and increased in-hospital mortality was significantly stronger in patients receiving high-dose glucocorticoids, as well as in those with diabetes or hypertension. These findings suggest that PNR may be particularly valuable in identifying high-risk individuals within already vulnerable subgroups. For instance, patients on high-dose steroids often present with blunted inflammatory responses, making traditional infection markers less reliable. In such cases, PNR could serve as a more nuanced indicator of immune dysregulation and disease severity. Similarly, the enhanced prognostic value observed in patients with metabolic comorbidities may reflect the compounding effects of chronic inflammation and endothelial dysfunction (35, 36), both of which influence PLT and NEUT dynamics. These interactions underscore the importance of integrating PNR into risk stratification models tailored to specific patient profiles. It is important to note that the subgroup findings for diabetes, hypertension, and high-dose glucocorticoid use, while based on formal statistical interaction tests, remain exploratory. They require validation in independent cohorts before any consideration for clinical application.

The novelty of our study lies in its focus on PNR as a composite marker in a specific and under-studied population—pneumonia patients receiving chronic or high-dose glucocorticoids. While previous literature has highlighted the prognostic role of individual hematologic parameters in pneumonia, our work was the first to evaluate the PNR in this context. PNR may help identify high-risk patients who could benefit from more aggressive monitoring, earlier escalation of care, or adjunctive immunomodulatory therapies. Furthermore, the significant interaction between PNR and comorbidities such as hypertension and diabetes suggests that PNR may be particularly useful in patients with metabolic conditions, who represent a substantial proportion of those receiving long-term glucocorticoids.

Despite these strengths, several limitations should be acknowledged. First, the retrospective design of the study limits the ability to establish causality and may introduce selection or information bias. Second, although we adjusted for a wide range of confounders, residual confounding cannot be entirely ruled out. A specific limitation is that inflammatory biomarkers such as C-reactive protein or procalcitonin were not routinely measured in the source study, with data missing for a large subset of patients. This precludes a direct comparison of PNR against these established markers within this cohort. Future prospective studies designed to measure both cellular ratios and protein-based biomarkers in all participants are needed to define their relative and combined prognostic utility. Third, the lack of granular data on glucocorticoid dosing regimens (e.g., oral vs. intravenous, tapering protocols) may limit the interpretation of immunosuppressive intensity. Fourth, our outcome was all-cause in-hospital mortality. The dataset did not allow for a distinction between deaths directly due to infectious complications (e.g., sepsis) and those from other causes (e.g., underlying disease progression or non-infectious complications), which could provide further mechanistic insight. Fifth, and most importantly, while PNR demonstrated good discriminative ability in our multicenter cohort, our findings require external validation in independent cohorts. Future prospective studies designed specifically to validate and refine the PNR are warranted.

In conclusion, our study provides preliminary evidence that the PNR is a promising prognostic marker in pneumonia patients receiving glucocorticoid therapy. A lower PNR is independently associated with increased in-hospital mortality risk and may help clinicians identify patients who require closer monitoring and more aggressive management. Future prospective studies are warranted to validate these findings, explore optimal cutoff values, and investigate whether PNR-guided interventions can improve clinical outcomes.

## Data Availability

The datasets presented in this study can be found in online repositories. The names of the repository/repositories and accession number(s) can be found at: the data that support the findings of this study are openly available in Dryad at https://doi.org/10.5061/dryad.mkkwh70x2.
