# Supplementary material for: Platelet-to-neutrophil ratio and in-hospital mortality in pneumonia patients receiving glucocorticoid therapy: a multicenter retrospective cohort study
Source: Front Med (Lausanne). 2026 Jan 12;12:1731128. doi: 10.3389/fmed.2025.1731128 (PMC12832381; doi:10.3389/fmed.2025.1731128)
Supplement: Supplementary file 1 [file Table_1.docx]

Supplementary Table S1 Univariate regression analysis of 30-day mortality in pneumonia patients receiving glucocorticoids

| Variables | *P* | HR (95%CI) |  |
| --- | --- | --- | --- |
|  |  |  |  |
| Gender (Male vs Female) | 0.455 | 0.885 (0.643 ~ 1.219) |  |
| Asthma | 0.167 | 0.250 (0.035 ~ 1.789) |  |
| COPD | **0.022** | 0.516 (0.292 ~ 0.910) |  |
| ILD | 0.164 | 1.253 (0.912 ~ 1.722) |  |
| Hypertension | 0.487 | 1.123 (0.809 ~ 1.560) |  |
| CHD | 0.883 | 1.037 (0.641 ~ 1.676) |  |
| CHF | 0.384 | 1.437 (0.635 ~ 3.251) |  |
| CRF | 0.984 | 0.994 (0.551 ~ 1.792) |  |
| Diabetes mellitus | 0.362 | 1.180 (0.827 ~ 1.684) |  |
| Nephrotic Syndrome | 0.320 | 1.253 (0.804 ~ 1.952) |  |
| CTD | 0.446 | 1.132 (0.823 ~ 1.557) |  |
| Cerebrovascular diseases | 0.102 | 0.506 (0.223 ~ 1.144) |  |
| Smoke |  |  |  |
| Never |  | 1.000 (Reference) |  |
| Former | 0.822 | 1.045 (0.713 ~ 1.531) |  |
| Current | **0.014** | 2.183 (1.173 ~ 4.063) |  |
| Alcoholism | 0.215 | 1.388 (0.827 ~ 2.330) |  |
| High dose glucocorticoid | **<0.001** | 2.135 (1.553 ~ 2.935) |  |
| Age (≥ 60 years) | 0.341 | 1.168 (0.848 ~ 1.609) |  |
| Temperature | **<0.001** | 1.319 (1.139 ~ 1.528) |  |
| Heartrate | **0.032** | 1.008 (1.001 ~ 1.015) |  |
| Systolic pressure | 0.416 | 1.003 (0.995 ~ 1.012) |  |
| Diastolic pressure | 0.385 | 0.994 (0.982 ~ 1.007) |  |
| LYM | **<0.001** | 0.646 (0.499 ~ 0.837) |  |
| HGB | 0.093 | 0.994 (0.988 ~ 1.001) |  |
| ALB | **<0.001** | 0.914 (0.890 ~ 0.939) |  |
| NUET | **<0.001** | 1.022 (1.011 ~ 1.033) |  |
| PLT | **<0.001** | 0.995 (0.993 ~ 0.997) |  |
| LDH | **<0.001** | 1.001 (1.001 ~ 1.001) |  |
| ALT | **0.003** | 1.003 (1.001 ~ 1.005) |  |
| TBIL | **0.054** | 1.004 (1.000 ~ 1.008) |  |
| CRE | **0.030** | 1.001 (1.001 ~ 1.003) |  |
| Pneumonia severity index | **<0.001** | 1.016 (1.012 ~ 1.020) |  |
| PNR |  |  |  |
| Tertile 1 |  | 1.00 (Reference) |  |
| Tertile 2 | **<0.001** | 0.452 (0.315 ~ 0.651) |  |
| Tertile 3 | **<0.001** | 0.215 (0.135 ~ 0.344) |  |

HR, Hazard Ratio; CI, Confidence Interval; COPD, Chronic Obstructive Pulmonary Disease; ILD, Interstitial Lung Disease; CHD, Coronary Heart Disease; CRF, Chronic Renal Failure; CTD, Connective Tissue Disease; NUET, Neutrophils; LYM, Lymphocytes; HGB, Hemoglobin; PLT, Platelets; ALB, Albumin; LDH, Lactate Dehydrogenase; ALT, Alanine Aminotransferase; TBIL, Total Bilirubin; CRE, Creatinine; PNR, Platelet to neutrophil ratio.
