# Supplementary material for: Platelet-to-neutrophil ratio and in-hospital mortality in pneumonia patients receiving glucocorticoid therapy: a multicenter retrospective cohort study
Source: Front Med (Lausanne). 2026 Jan 12;12:1731128. doi: 10.3389/fmed.2025.1731128 (PMC12832381; doi:10.3389/fmed.2025.1731128)
Supplement: Supplementary file 2 [file Table_2.docx]

Supplementary Table S2 Univariate regression analysis of 90-day mortality in pneumonia patients receiving glucocorticoids

| Variables | *P* | HR (95%CI) |  |
| --- | --- | --- | --- |
|  |  |  |  |
| Gender (Male vs Female) | 0.276 | 0.847 (0.629 ~ 1.141) |  |
| Asthma | 0.122 | 0.212 (0.030 ~ 1.517) |  |
| COPD | **0.020** | 0.544 (0.326 ~ 0.910) |  |
| ILD | **0.015** | 1.446 (1.075 ~ 1.945) |  |
| Hypertension | 0.429 | 1.131 (0.834 ~ 1.535) |  |
| CHD | 0.529 | 1.148 (0.746 ~ 1.766) |  |
| CHF | 0.055 | 1.928 (0.986 ~ 3.771) |  |
| CRF | 0.985 | 1.005 (0.582 ~ 1.735) |  |
| Diabetes mellitus | 0.145 | 1.274 (0.920 ~ 1.764) |  |
| Nephrotic Syndrome | 0.482 | 1.164 (0.762 ~ 1.777) |  |
| CTD | 0.245 | 1.193 (0.886 ~ 1.605) |  |
| Cerebrovascular diseases | 0.130 | 0.578 (0.285 ~ 1.175) |  |
| Smoke |  |  |  |
| Never |  | 1.00 (Reference) |  |
| Former | 0.618 | 1.093 (0.770 ~ 1.552) |  |
| Current | **0.013** | 2.112 (1.167 ~ 3.823) |  |
| Alcoholism | 0.363 | 1.261 (0.765 ~ 2.080) |  |
| High dose glucocorticoid | **<0.001** | 2.155 (1.603 ~ 2.897) |  |
| Age (≥ 60 years) | 0.075 | 1.313 (0.973 ~ 1.772) |  |
| Temperature | **<0.001** | 1.345 (1.175 ~ 1.540) |  |
| Heartrate | 0.102 | 1.006 (0.999 ~ 1.012) |  |
| Systolic pressure | 0.533 | 1.002 (0.995 ~ 1.010) |  |
| Diastolic pressure | 0.175 | 0.992 (0.980 ~ 1.004) |  |
| LYM | **<0.001** | 0.662 (0.522 ~ 0.839) |  |
| HGB | **0.028** | 0.993 (0.987 ~ 0.999) |  |
| ALB | **<0.001** | 0.921 (0.898 ~ 0.944) |  |
| NUET | **<0.001** | 1.022 (1.012 ~ 1.033) |  |
| PLT | **<0.001** | 0.996 (0.994 ~ 0.998) |  |
| LDH | **<0.001** | 1.001 (1.001 ~ 1.001) |  |
| ALT | **<0.001** | 1.003 (1.001 ~ 1.005) |  |
| TBIL | **0.062** | 1.004 (1.000 ~ 1.007) |  |
| CRE | 0.072 | 1.001 (1.000 ~ 1.002) |  |
| Pneumonia severity index | **<0.001** | 1.017 (1.013 ~ 1.021) |  |
| PNR |  |  |  |
| Tertile 1 |  | 1.00 (Reference) |  |
| Tertile 2 | **<0.001** | 0.440 (0.314 ~ 0.617) |  |
| Tertile 3 | **<0.001** | 0.203 (0.131 ~ 0.315) |  |

HR, Hazard Ratio; CI, Confidence Interval; COPD, Chronic Obstructive Pulmonary Disease; ILD, Interstitial Lung Disease; CHD, Coronary Heart Disease; CRF, Chronic Renal Failure; CTD, Connective Tissue Disease; NUET, Neutrophils; LYM, Lymphocytes; HGB, Hemoglobin; PLT, Platelets; ALB, Albumin; LDH, Lactate Dehydrogenase; ALT, Alanine Aminotransferase; TBIL, Total Bilirubin; CRE, Creatinine; PNR, Platelet to neutrophil ratio.
